# Facial color matching in optical see-through augmented reality

**DOI:** 10.1167/jov.25.10.16

**Published:** 2025-08-28

**Authors:** Yanmei He, Christopher A. Thorstenson

**Affiliations:** 1 Munsell Color Science Laboratory, Rochester Institute of Technology, Rochester, NY, USA

**Keywords:** face color, skin tone, OST-AR, augmented reality, ambient lighting

## Abstract

Augmented reality (AR) aims to combine elements of the surrounding environment with additional virtual content into a combined viewing scene. Displaying virtual human faces is a widespread practical application of AR technology, which can be challenging in optical see-through AR (OST-AR) because of limitations in its color reproduction. Specifically, OST-AR's additive optical blending introduces transparency and color-bleeding, which is exacerbated especially for faces having darker skin tones, and for brighter and more chromatic ambient environments. Given the increasing prevalence of social AR applications, it is essential to better understand how facial color reproduction is impacted by skin tone and ambient lighting in OST-AR. In this study, a psychophysical experiment was conducted to investigate how participants adjusted colorimetric dimensions of OST-AR-displayed faces to match the color of the same faces viewed on a conventional emissive display. These adjustments were made for faces having six different skin tones, while under different simulated ambient luminance (“low” vs. “high”) and chromaticity (warm, neutral, cool). Additionally, participants rated their adjustments for overall appearance match and preference. The results indicate that the magnitude and specific dimensions of colorimetric adjustments needed to make matches varied across skin tones and ambient conditions. The current work is expected to facilitate virtual human face reproduction in AR applications and to foster more equitable and immersive extended reality environments.

## Introduction

Augmented reality (AR) aims to enhance how we experience and interact with our surroundings by integrating virtual content with our perception of the real world. AR combines digital objects, information, and interactive elements together with aspects of our surrounding physical environment, allowing virtual content to appear as though it exists within that same environment. One of the current paradigms used to implement AR experiences is optical see-through augmented reality (OST-AR), which is increasingly used across automotive, medical, entertainment, education, and manufacturing domains (Nee & Ong, 2023; Xiong, Hsiang, He, Zhan, & Wu, 2021). OST-AR operates on an additive light model, integrating virtual content with the physical environment via a transparent optical combiner, allowing users to perceive both simultaneously. However, OST-AR has limitations in color reproduction because of its additive light blending. Specifically, this blending introduces transparency whereby color from the surrounding environment “bleeds through” the virtual content. This is particularly problematic for the AR system when producing darker colors, and when viewed in brighter and more complex ambient environments (Chapiro, Kim, Asano, & Mantiuk, 2024; Erickson, Kim, Bruder, & Welch, 2020; Liu, Jindal, Mantel, Forchhammer, & Mantiuk, 2022).

Displaying virtual faces is integral across many AR applications—including in marketing (Javornik, Marder, Pizzetti, & Warlop, 2021), social interaction (Hirskyj-Douglas et al., 2020; Javornik et al., 2022), remote collaboration (Osmers et al., 2021), telecommunication (Maimone et al., 2013; Wang, Wang, Shi, Zhang, & Zheng, 2020; Wang, Ye, Sandor, Zhang, & Fu, 2022), entertainment (O'Meara & Szita, 2023), healthcare (Borresen et al., 2019), and education (Wedyan et al., 2021; Zsigmond & Buhai, 2021).

However, OST-AR's challenges with color reproduction extend considerably when rendering virtual faces, particularly those having darker skin tones, and when viewed in brighter ambient lighting. These challenges can reduce realism and engagement, diminish usability, lead to misrepresentation, and introduce potential racial bias in these systems (Benjamin et al., 2023; Itoh, Langlotz, Sutton, & Plopski, 2022; Peck, Good, Erickson, Bynum, & Bruder, 2022). Therefore it is essential to better understand how to optimize OST-AR color reproduction toward displaying virtual faces. The current research aims to evaluate the colorimetric factors that contribute to limitations in facial color reproduction in OST-AR, with a focus on how these might vary as a function of skin tone, ambient luminance, and ambient chromaticity.

Facial appearance is capable of conveying myriad social information about a person and influences how we perceive and interact with them in social, professional, and legal contexts (Zebrowitz & Montepare, 2008). In particular, the appearance of facial skin color has been shown to inform perceptions of attractiveness, healthiness, youthfulness, and emotion (Benitez-Quiroz et al., 2018; Fink, Bunse, Matts, & D'Emiliano, 2012; Fink, Grammer, & Matts, 2006; Stephen, Law Smith, Stirrat, & Perrett, 2009; Thorstenson et al., 2018; Thorstenson et al., 2022). Further, past research has found that perception and memory for facial skin color is particularly heightened relative to non-face objects (Hasantash, Lafer-Sousa, Afraz, & Conway, 2019; Tan & Stephen, 2013; Thorstenson, Pazda, & Elliot, 2017), and that people are particularly sensitive to poor reproduction of skin color (Fernandez & Fairchild, 2001; Zeng & Luo, 2013). Therefore optimizing the color appearance of facial skin is essential toward improving intended experiences in OST-AR environments.

Rendering virtual faces in OST-AR is particularly challenging because facial skin tones vary naturally across the human population. Physiologically, skin tone is determined largely by melanin chromophores in the skin, influencing how it absorbs, scatters, and reflects light (Duteil, Roussel, & Bahadoran, 2017; Jablonski & Chaplin, 2000; Zonios, Bykowski, & Kollias, 2001). Melanin acts as a photoprotective filter by reducing light penetration into subepidermal tissues. Melanin concentration in humans was shaped as an adaptation to differing geographic UV radiation pressures (Jablonski & Chaplin, 2000), with melanosomes densely packed and evenly distributed in darker skin while less abundant in lighter skin (Kaidbey, Agin, Sayre, & Kligman, 1979). Recent work has demonstrated substantial disparities in OST-AR facial skin tone reproduction. Because OST-AR generally operates by ‘adding’ virtual light to our perception of the surrounding environment, virtual faces having darker skin tones add minimal extra light and are thus perceived as more transparent than those having lighter skin tones. This transparency can lead to people with darker skin being dehumanized (Peck et al., 2022), and needing greater brightness compensations to be perceived as commensurately transparent to lighter facial skin (Herbeck, Murdoch, Thorstenson, Murdoch, & Thorstenson, 2024). Brighter ambient conditions can also decrease perceptions of social presence for OST-AR avatars (Benjamin et al., 2023). In particular, Doroodchi and colleagues (2022) found that when customizing self-avatars in OST-AR by selecting among 10 skin color options, participants with darker (but not lighter) skin had to select lighter skin colors when under brighter ambient lighting to adequately represent their own appearance. Altogether, these findings demonstrate limits in OST-AR facial color reproduction, and how they are disproportionally impacted by skin tone and ambient luminance. The current study seeks to extend this work by investigating OST-AR facial color reproduction as a function of multiple independent colorimetric dimensions, a systematically diverse range of skin tones, and under varied ambient lighting conditions including luminance and chromaticity.

The current study examines how skin tone and ambient lighting affect facial color reproduction in OST-AR. First, participants were asked to adjust the color of AR-displayed face models to match the color of the same face models viewed on a conventional emissive display (“color-matching task”). Participants could adjust the color of the AR faces along CIELAB dimensions of L* (lightness), C* (chroma), and h° (hue angle), independently. C* and h° are derived from the a* and b* components of CIELAB by converting from Cartesian to polar coordinates, where C* = √(a*² + b*²) and h° = arctan (b*/a*). This representation allows for more perceptually meaningful adjustments of chroma and hue. Participants made these adjustments for models having six different skin tones (derived from the Monk Skin Tone Scale), and under variable ambient lighting conditions simulated by the emissive display, including variations in luminance (“black,” “low,” “high”) and chromaticity (warm, neutral, cool). This task aimed to evaluate the extent to which each colorimetric dimension impacted facial color appearance in OST-AR, and how these varied as a function of ambient lighting and skin tone. We anticipated that lightness adjustments would vary largely as a function of skin tone and ambient luminance, whereas chroma or hue adjustments might play a larger role across changes in ambient chromaticity. In two subsequent tasks, we presented participants with the AR faces having the same appearance as their adjustments made during the color-matching task, so that they could re-evaluate the same stimuli corresponding to their own responses. Participants then rated the extent to which their AR adjustments actually matched the appearance of the emissive stimuli (“match-rating task”), and the extent to which they were visually appealing (“preference-rating task”). Based on prior research, we anticipated faces having darker skin tones would exhibit less accurate and less preferred matches, particularly in higher ambient luminance conditions. Altogether, these tasks provide nuanced information about how participants modify facial color in OST-AR to appear optimal as possible, yet identifies limitations with respect to their resulting apperance.

## Methods

### Participants

A total of 27 volunteers from Rochester Institute of Technology participated in the experiment. Two participants were excluded from subsequent analyses: one due to color vision deficiency (exhibited by Ishihara screening and self-report), and one due to inattention (exhibited by unreasonably rapid responses noted by the experimenter). Data from the remaining 25 participants (Age_Mean_ = 26.96, Age_SD_ = 5.15; 9 males, 15 females, one non-binary; 11 Asian, nine White, one Black or African American, one Native Hawaiian or Pacific Islander, one multiracial, and two identifying as “other”) were used in the final analyses. The experimental protocol was approved by the Human Subjects Research Office at Rochester Institute of Technology.

### Experimental setup

**Figure 1.**
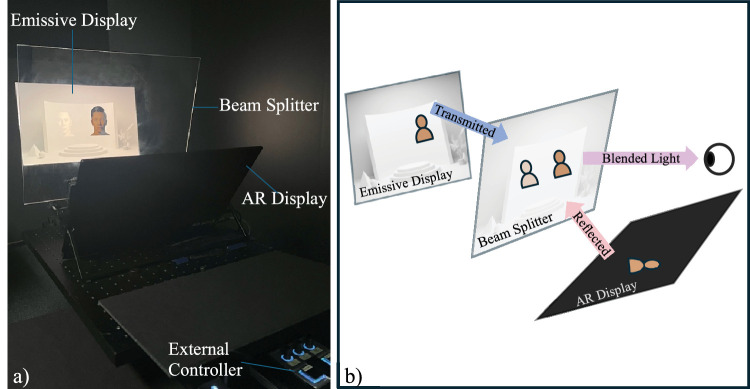
(**a**) Photograph of the OST-AR setup. (**b**) Diagram of the setup.

A desktop OST-AR setup consisting of a 27 in. DELL UP2720Q IPS LCD monitor (referred to as the “emissive display”), an 18-inch InnoView PM808-01B IPS LCD monitor (referred to as the “AR display”), and a 14-inch × 18-inch glass beam splitter was used in the experiment (see Figure 1). The emissive display was upright, and the AR display tilted at ∼35° from vertical. The beam splitter (63% transmittance, 35% reflectance) was placed between them, tilted 12° from vertical, at distances of 30 cm and 25 cm from the emissive and AR displays, respectively. Both displays had a resolution of 2560 × 1600. The observer's viewing position was approximately 62 cm from the beam splitter. The emissive display was viewed directly through the beam splitter (and was meant to simulate a “real” surrounding environment), whereas the AR display's light was reflected by the beam splitter (and was used to introduce additional virtual content). The equipment was positioned such that the AR stimuli were perceived to be co-planar with the emissive content. The emissive and AR displays were controlled by a PC running Windows 10 and MATLAB R2023a. The experiment was conducted in a dark room with all other lights off.

We characterized both displays with the goal of ensuring colorimetrically accurate matches between AR and emissive stimuli, before ambient complexity was added (i.e., when the emissive background was completely black). Using display colorimetric measurements by a CR-250 spectroradiometer through the beam splitter, two gamma-offset-gain (GOG) models were established to map color values between device RGB space and CIE 1931 XYZ space (Berns, 1996; Fairchild & Wyble, 1998). Verification showed good performance between measured and model-predicted XYZ values (ΔE_00_emissiveMean_ = 0.18, ΔE_00_emissiveMax_ = 0.55; ΔE_00_ARMean_ = 0.17, ΔE_00_ARMax_ = 0.38). Therefore these models were used to process the stimuli when shown on their respective displays.

### Stimuli

#### Skin tones

**Figure 2.**
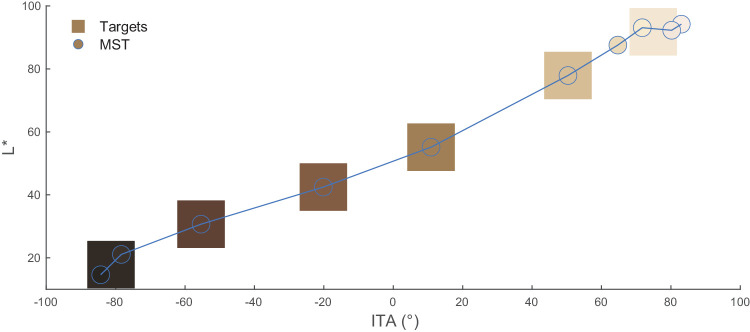
Target skin tones (squares) used in the current experiment, shown in the L* versus ITA plane. MST skin tones (circles connected by line) shown as reference.

We first selected target skin tones to capture the broad range of skin tones among the human population while reducing these to be reasonably feasible for the experimental tasks. The 10-shade Monk Skin Tone Scale (MST) was designed to represent the broad range of skin tones for skin tone classification among a diverse population (Monk, 2023). We calculated CIELAB L* (perceptual lightness) and individual typology angle (ITA) for each MST skin tone (see Figure 2). ITA is defined as an angle using the CIELAB L*-b* plane, approximating melanin content in the skin (Chardon, Cretois, & Hourseau, 1991). We used ITA in the current study to denote skin tone variation due to its linear correlation with melanin concentration (Zonios et al., 2001), and we used CIELAB L* because of expected perceptual disparities in displaying AR content with varying lightness. To ensure a broad range of skin lightness and melanin while avoiding redundant visual appearance, we derived six distinct skin tones as our target skin tones from the MST (see Figure 2 and Table 1). These six target skin tones were then used to generate the face stimuli.

**Table 1.** Colorimetric and ITA values for target skin tones.

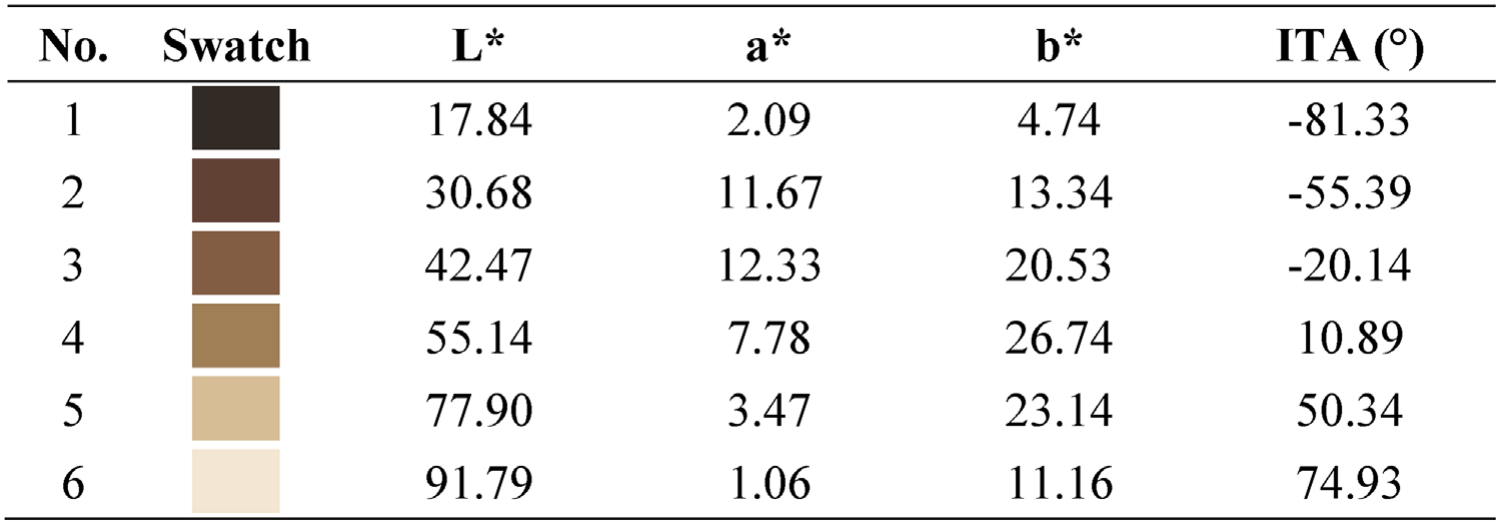

#### Faces

**Figure 3.**
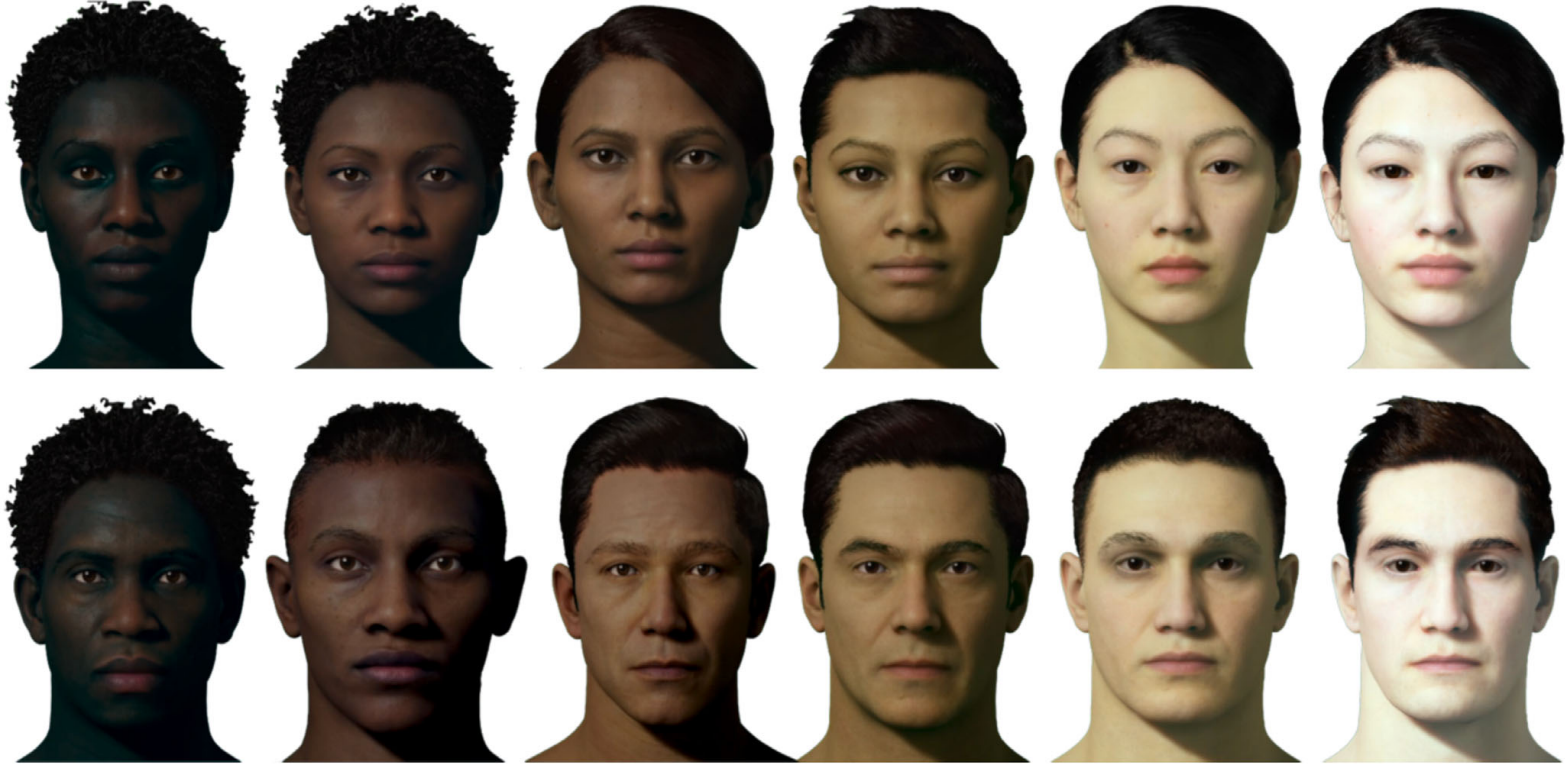
Twelve faces spanning six skin tones were used in the current experiment.

We then selected 12 virtual avatars (six male, six female) from pre-generated MetaHuman (Epic Games, 2021) models approximating our target values. Finally, we computed image statistics for the diffuse skin areas of the rendered models and further modified their color appearance (using MATLAB), such that the average skin tone for the models more precisely matched our target colors (see Figure 3).

#### Ambient lighting conditions

We selected nine ambient lighting conditions, comprising differing combinations of peak luminance (Y) and correlated color temperature (CCT), which were used to generate background scenes (emissive) and face stimuli (emissive and AR) to simulate their appearance under these ambient conditions.

**Figure 4.**
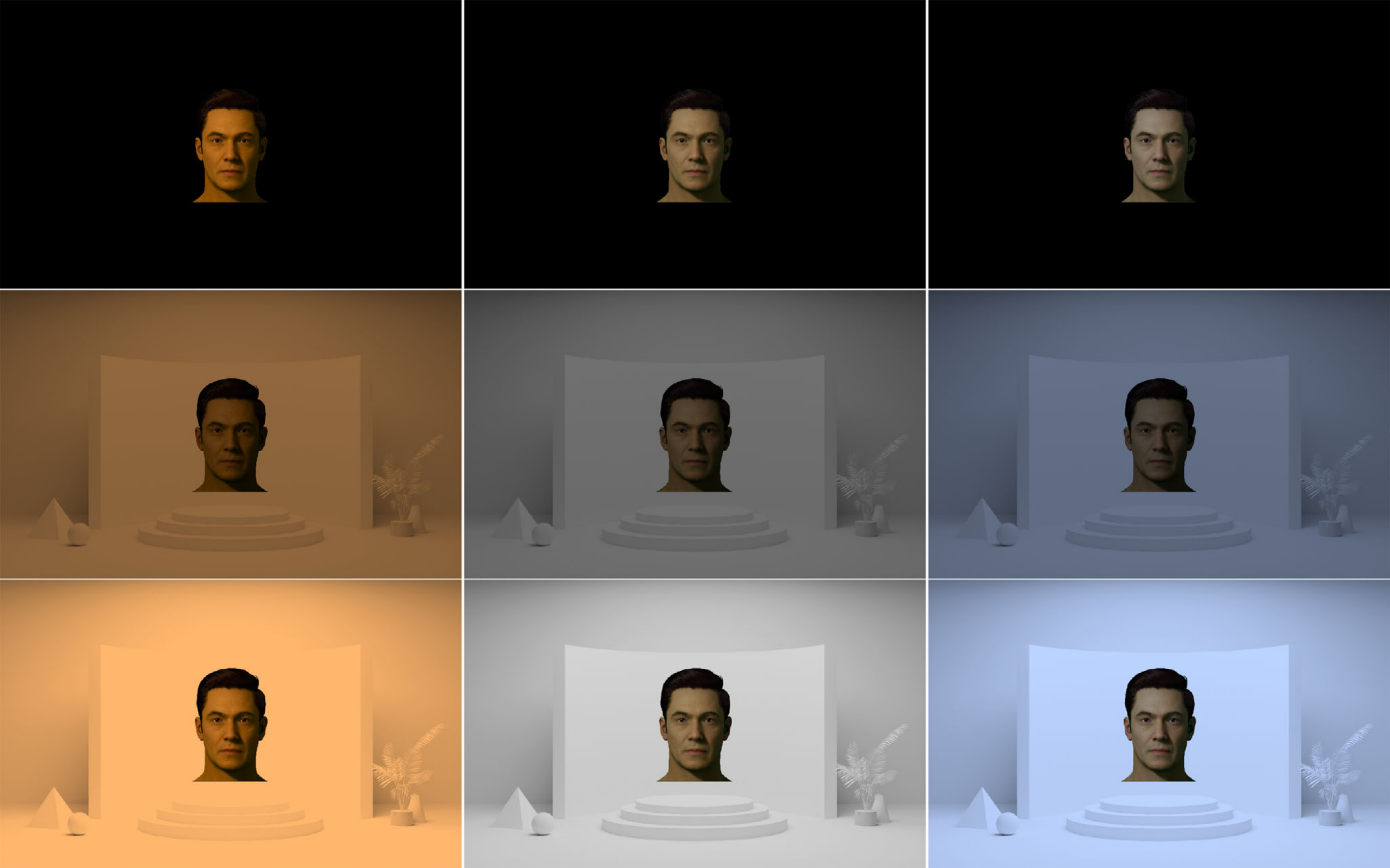
Example of the nine ambient lighting conditions used in the current experiment. The rows from top-to-bottom show black (control), low, and high luminance conditions. The columns from left to right show warm, neutral, and cool CCT conditions.

A background scene was generated using Blender (Blender Foundation, 2024). The scene was chromatically neutral, had a bright center wall for positioning facial stimuli, and was diffusely lit with predictable shadows, enabling reliable estimation about the ambient lighting. The scene had an average luminance of 64%, and a maximum luminance of 84%, of the reference white point. We then modified the appearance of this background scene (and the face stimuli within each scene) to simulate the multiple ambient lighting conditions, comprising combinations of peak luminance (“low” vs. “high”) and chromaticity (“warm,” “neutral,” “cool”; See Figure 4 and Table 2).

**Table 2.** Nine ambient conditions used in the current experiment. *Notes*: Luminance (Y) and CCT values correspond to the simulated ambient lighting conditions used to render the background and foreground stimuli.

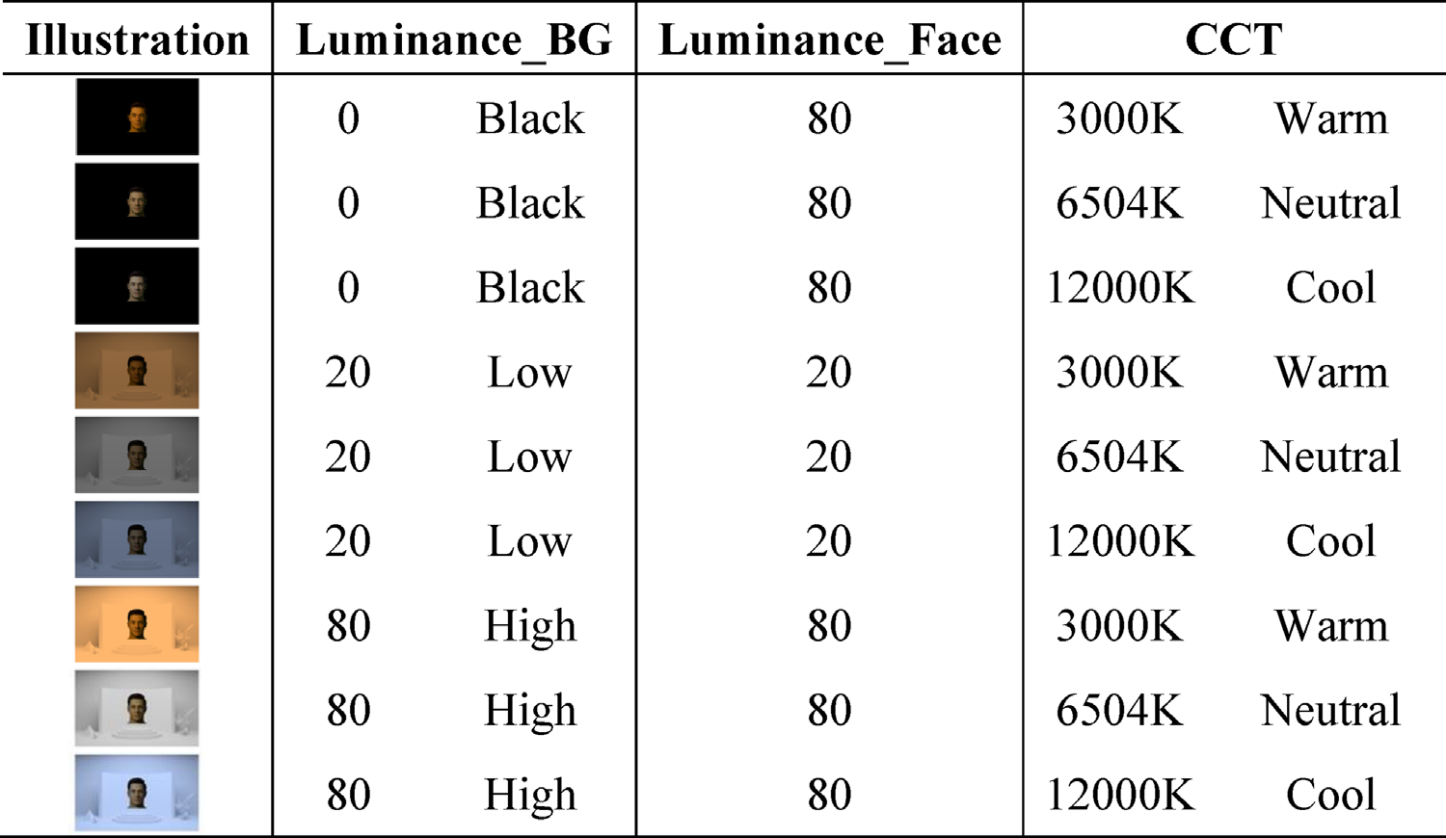

**Table 3.** Mean color differences (ΔE₀₀) between adjusted AR face colors and their emissive counterparts for the stimuli shown in Figure 13. *Notes*: Each column group corresponds to one of the three face stimuli (light, mid, and dark skin tones, from left to right), and each cell reflects the mean ΔE₀₀ for a specific ambient luminance (rows: Black, Low, High) and ambient CCT (columns: Warm, Neutral, Cool).

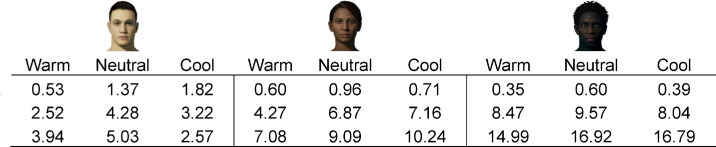

The ambient condition simulations were implemented by applying varied white-point estimates to stimuli using the CAT16 chromatic adaptation transformations (CAT) (Li et al., 2017; Peng, Cao, Zhai, & Luo, 2021). The two peak luminance conditions were simulated as having light sources of Y = 20 cd/m² (“low”) and Y = 80 cd/m² (“high”). These values were chosen based on joint considerations of the effective peak luminances of both emissive and AR displays (i.e., light transmitting through or reflecting from the beam splitter), and of gamut constraints for both displays when applying the CATs to stimuli.

The three chromaticity conditions were simulated as having light sources with CCTs of 3000K (“warm”), 6504K (“neutral”), and 12000K (“cool”). These values were selected to represent natural light variations along the Planckian locus, encompassing the yellowish light of sunrise/sunset, neutral average daylight, and the bluish light of the north skylight (Hernández-Andrés, Lee, & Romero, 1999; Judd et al., 1964).

Finally, we additionally included ambient conditions whose backgrounds were completely black, where no light from the emissive background scene affected the AR stimuli rendering. These served as control conditions to evaluate the extent to which skin color matches could be made without considering the complex factors introduced by variable ambient lighting conditions. In this condition, the luminance of the faces was made to be the same as in the “high” luminance condition, to provide the highest amount of contrast. In total, there were nine ambient conditions in this study (see Figure 4 and Table 2).

### Procedure

Participants entered the laboratory, signed a consent form, and completed an initial color vision deficiency screening using a short form of the Ishihara test. After screening, they were given practice trials to familiarize themselves with the controller functions and task instructions. Participants were asked to complete three separate tasks: a “color-matching” task, a “match-rating” task, and a “preference-rating” task.

#### Color-matching task

**Figure 5.**
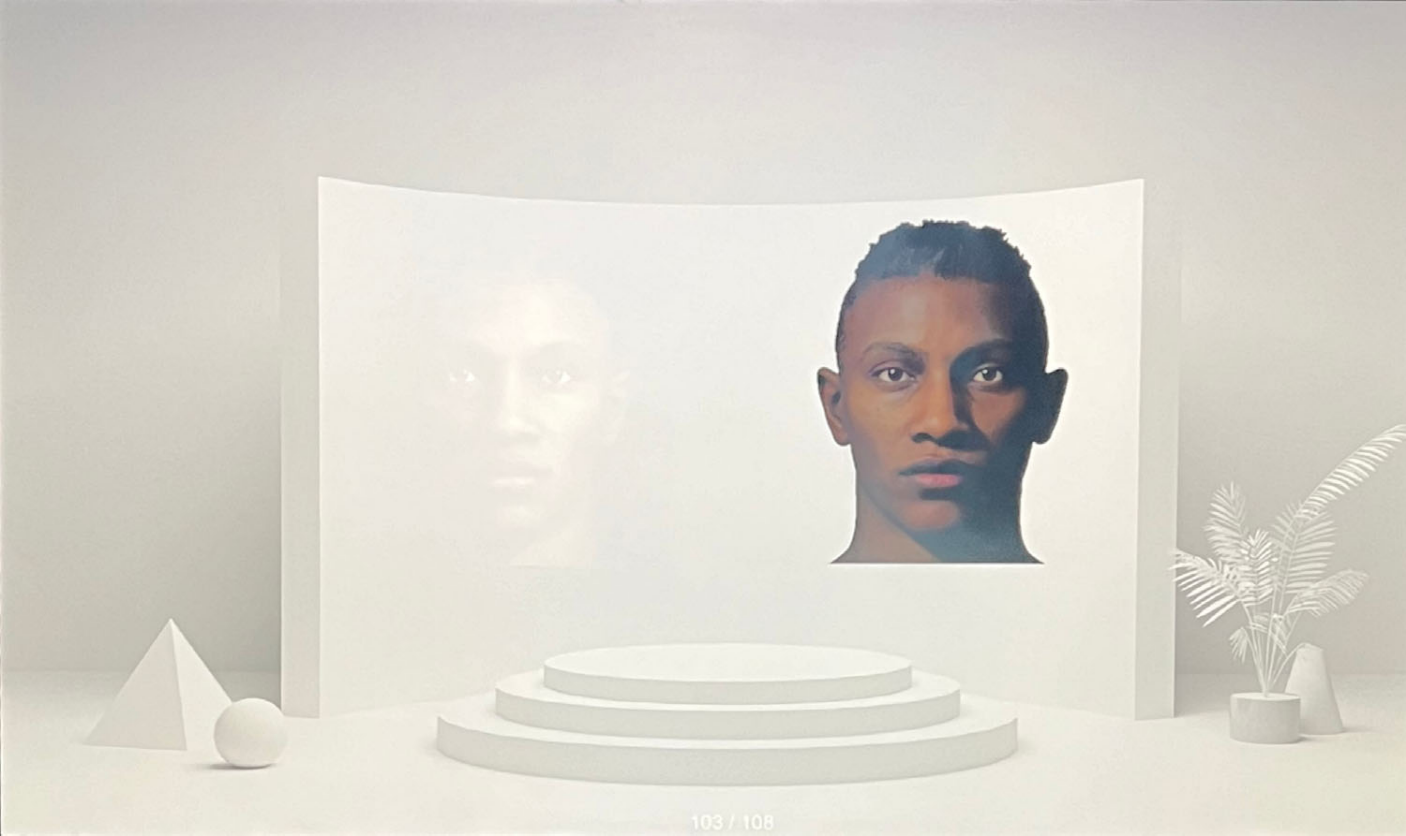
Sample trial for the color-matching task. Participants were asked to adjust the color of the AR face (left) to best match the appearance of the emissive face (right).

In the color-matching task, each trial presented participants with a background scene and two faces: one generated by the emissive display (right) and the other by the AR display (left; See Figure 5). The background scene and both faces were aligned with a specific ambient condition, and the facial color on the AR display was initially calibrated to match that of the emissive face, based on matched colorimetric calibration using GOG display models and the same chromatic adaptation transformation (CAT16) applied to the face stimuli on both displays. Participants were instructed to adjust the color of the AR face image along the L* (lightness), C* (chroma), and h° (hue) dimensions, to best match the appearance of the emissive face, using three physical knobs on an external controller. The knobs adjusted each colorimetric parameter separately, but participants could modify all three within a single trial. Each colorimetric adjustment was applied globally for each pixel in the AR face image.

For the L* adjustment, modifying the lightness gamma (the exponent in a power-law function) was found to be an effective approach for OST-AR displays, as it enhances visibility and contrast in dark areas while having a proportionally smaller impact on bright areas, especially when γ < 1 (Herbeck et al., 2024; Lee & Kim, 2018). In the current work, participants adjusted the gamma exponent on the L* channel for pixels in the AR face image using a step size of 0.02, within a range with a lower bound of -1 and an unconstrained upper bound (which did not exceed 1.04 in participant responses). Notably, γ = 1 (the default value with no gamma exponent adjustment) corresponds to the unadjusted AR face image. C* adjustments were made to the AR face image pixels with a step of 0.5 and a range adapted to the ambient condition, ensuring that the adjusted C* values for all target skin tones remained between 0 and 100. Adjustments to the h° (hue) values for pixels in the AR face image were performed with a step of 1 and a range from −45° to 45°. While making the color-matching adjustments, participants were asked to primarily focus on matching the skin areas of the face rather than other areas such as hair, eyes, or lips.

**Figure 6.**
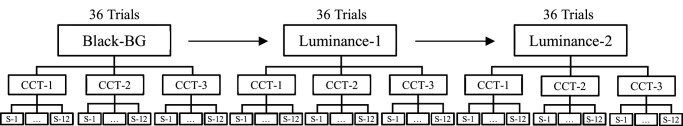
Overview of trials for the color-matching task. Top layer for background conditions: the black background and two luminance (“high” or “low”) conditions; middle layer for the three CCTs (3000K, 6504 K, or 12,000 K); bottom layer for the 12 face stimuli.

Participants always completed trials with the black background first, as these served as the baseline conditions without background complexity. In these trials, CCT conditions were presented in blocks, with the CCT block order randomly selected, and faces presented randomly within blocks (36 trials). Following these, complexity was added with the background scene that had one of two luminance levels (“high” vs. “low”) and one of three CCTs (corresponding to “warm,” “neutral,” and “cool”), with the faces rendered under the same conditions as the background. In these trials, luminance and CCT conditions were also presented in blocks. The order of luminance blocks was randomized, and the CCT block order was randomized within the luminance blocks. Face stimuli were presented randomly within CCT blocks (72 trials). A 20-second adaptation period was provided once before all trials with the black background scenes, as well as at each transition of luminance or CCT for the more complex background scenes. During the adaptation period, only the new background was visible, and participants were instructed to simply look around the scene for the full 20 seconds, and do nothing else. There were 108 total trials for the color-matching task (see Figure 6 for an example of the trial order structure).

#### Match-rating task

**Figure 7.**
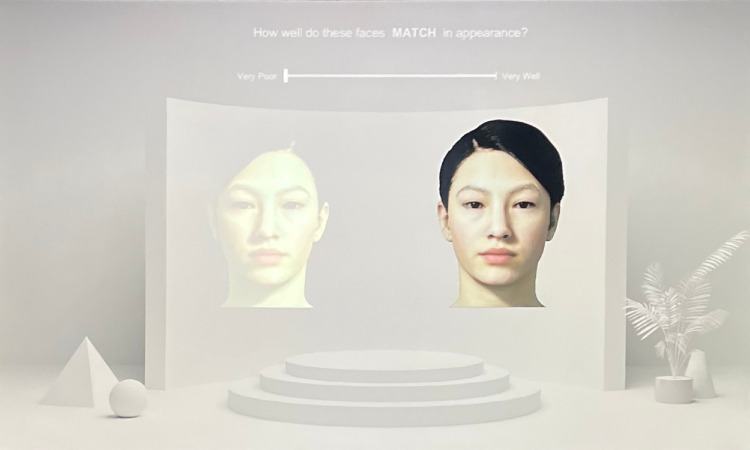
Sample trial for the match-rating task. Participants were shown their AR adjustments from the previous color-matching task (left) and were asked to rate how well it matched the emissive face (right).

In the match-rating task, the matches generated by participants during the previous color-matching task were shown again in the same order for participants to evaluate how well their adjusted faces matched the appearance of the emissive display face (see Figure 7). This task was presented in the same way as in the previous color-matching task, but with an added text prompt (“How well do these faces MATCH in appearance?”), along with a digital slider for participants to provide their ratings (0 “very poor” to 100 “very well”), for a total of 108 trials.

#### Preference-rating task

**Figure 8.**
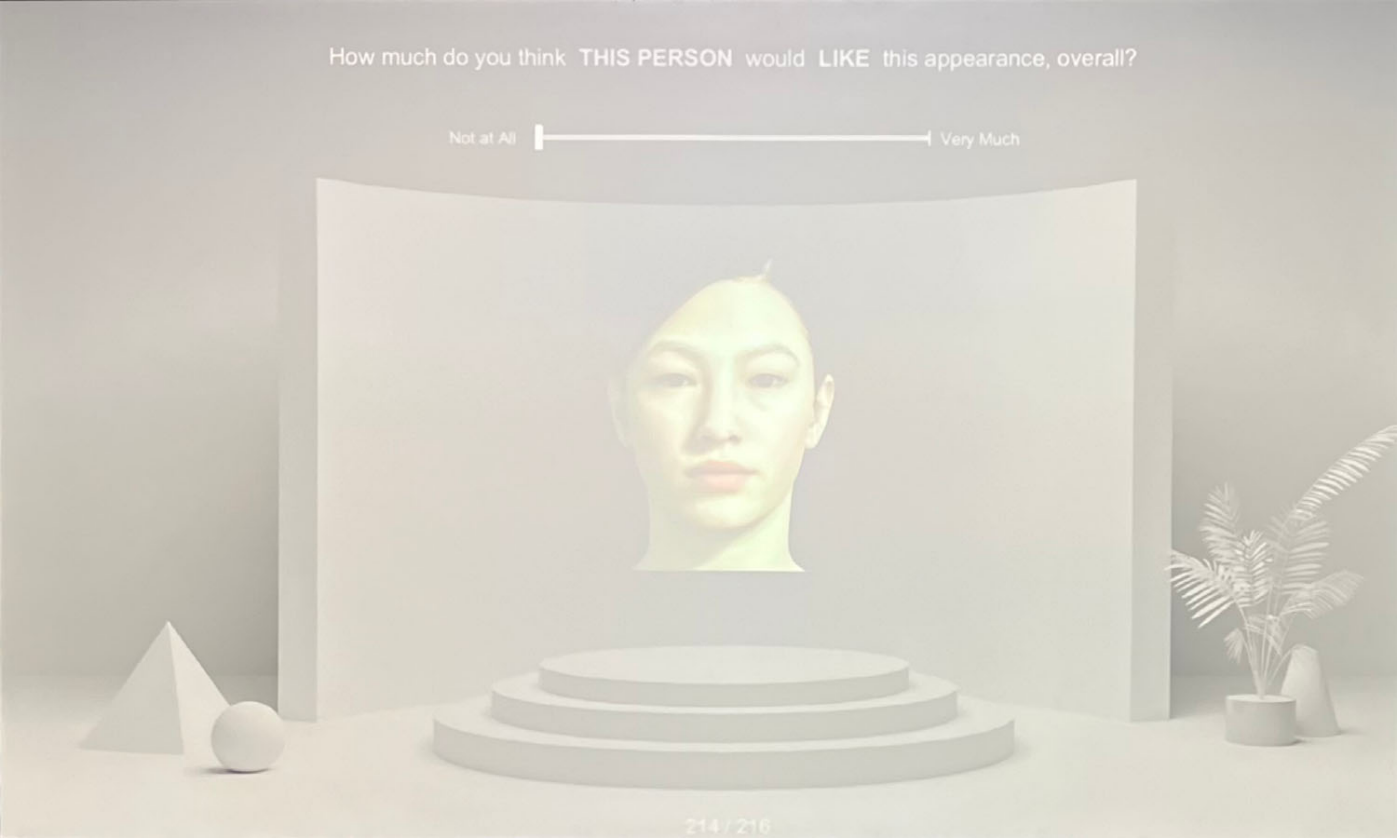
Sample trial for the preference-rating task. Participants were shown their AR adjustments from the color-matching task and were asked to rate perceived preference for its appearance.

In the preference-rating task, the matches from the color-matching task were presented once again in the same order for participants to assess how well they generally appeared. For this task, only the AR face (without the emissive face) was shown (see Figure 8). A text prompt (“How much do you think THIS PERSON would LIKE this appearance, overall?”) was presented with a slider to provide their ratings (0 “not at all” to 100 “very much”). This particular wording of the question was chosen to elicit ratings about preference for the general appearance of the stimuli, rather than provoke participant attitudes based on stimulus identity (e.g., to avoid making inferences based on perceived attractiveness, etc.). This task had 108 trials, for a total of 324 trials across the entire experiment. Participants were encouraged to take short breaks in between each task, and generally completed the experiment in approximately one hour.

## Results

### Color-matching task

**Figure 9.**
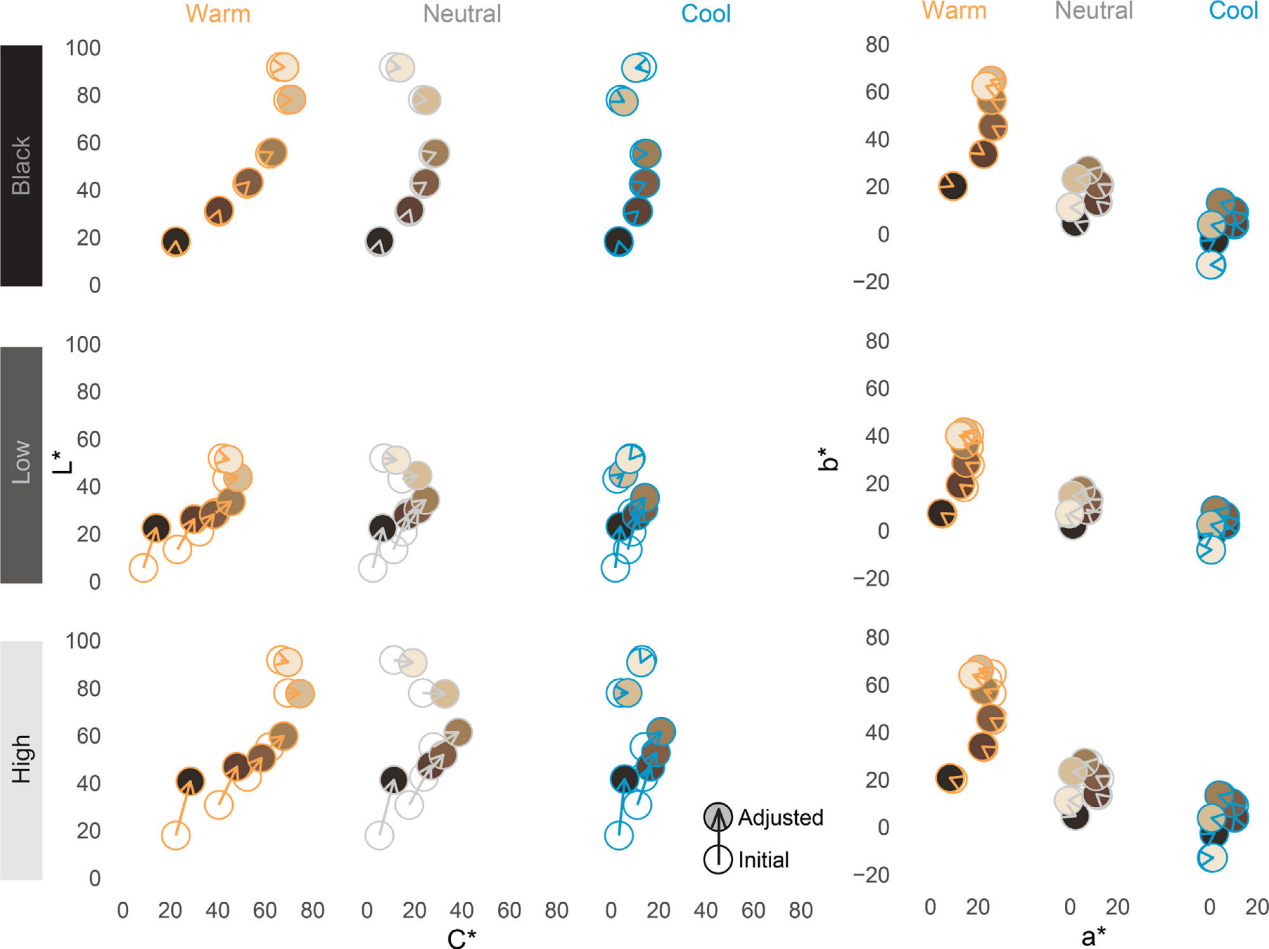
Initial versus adjusted AR skin color to match the appearance of emissive faces in the color-matching task. Color adjustments were negligible in the black background (control) conditions, but were more evident with added ambient complexity. Left: Lightness (L*) and Chroma (C*) adjustments varied widely as a function of ambient luminance (low vs. high), ambient CCT (warm-neutral-cool), and skin tone. Right: Hue adjustments (ΔH*; showing changes in h° after controlling for changes in C*, plotted in a* x b* plane) were largely negligible across most ambient conditions, but with some hue adjustments becoming evident in the high luminance, warm CCT ambient condition.

Under the black background conditions, participants made negligible adjustments to lightness (ΔL*; *M* = 0.22, *SE* = 0.15), chroma (ΔC*; *M* = 0.70, *SE* = 0.16), and hue (ΔH*; *M* = 0.37, *SE* = 0.09) to match between AR and emissive face appearance. This suggests that participants had no difficulty matching appearance under the control condition, and that the challenges in matching appearance under other conditions were due to the added ambient complexity (see Figure 9 and Figure 13). For the rest of this section, we further evaluate the patterns of color adjustments participants made to AR faces to match emissive faces under those ambient conditions.

Repeated-measures analyses of variance (ANOVAs) were conducted to evaluate the extent to which participants adjusted AR facial lightness (ΔL∗), chroma (ΔC∗), and hue (ΔH*) to optimize their appearance matches to emissive faces. The independent variables included skin tone (6), ambient luminance (2; low vs. high), and ambient CCT (3; warm, neutral, cool). The dependent variables were ΔL∗, ΔC∗, and ΔH*.

#### Lightness

There was a significant effect of skin tone on ΔL*, *F*(5, 120) = 67.48, *p* < 0.001, indicating that participants had to increase lightness more as skin tones became darker. There was a significant effect of ambient luminance on ΔL*, *F*(1, 24) = 8.60, *p* = 0.007, indicating that participants generally had to increase lightness more in high luminance (*M* = 8.87, *SE* = 1.53) than low luminance (*M* = 7.65, *SE* = 1.59) ambient conditions. There was no significant effect of ambient CCT on ΔL*, *F*(2, 48) = 2.80, *p* = 0.071, suggesting that ambient lighting chromaticity did not impact lightness adjustments needed to match AR to emissive face appearance.

**Figure 10.**
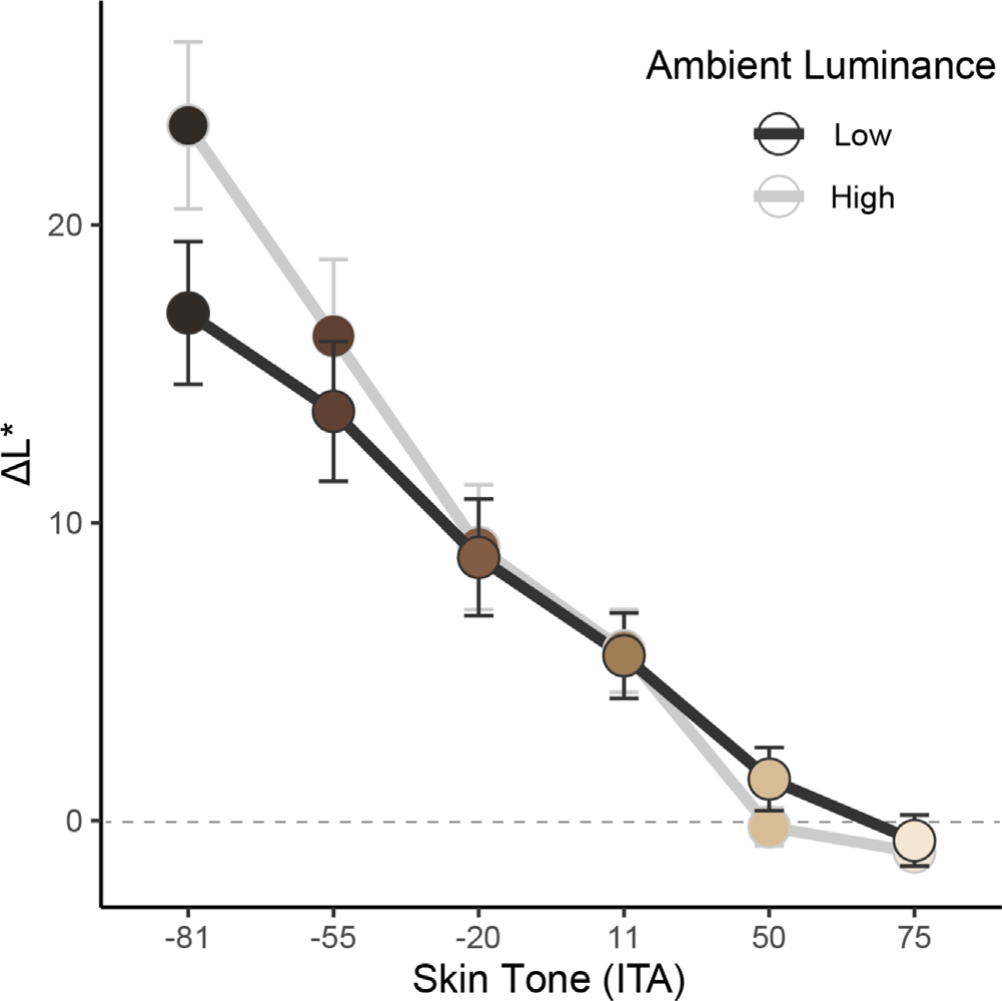
Summary (mean and SE) of the influence of skin tone (ITA) and ambient luminance (low vs. high) on lightness adjustments made to AR faces to match the appearance of emissive faces in the color-matching task. Darker skin tones needed more lightness adjustments than lighter skin tones, particularly under high ambient luminance conditions.

There was also a significant interaction between skin tone and ambient luminance on ΔL*, *F*(5, 120) = 20.36, *p* < 0.001, indicating that the influence of skin tone on ΔL* varied as a function of ambient luminance. This interaction indicated that darker skin tones required greater lightness adjustments under high ambient luminance compared to low ambient luminance, and that this difference diminished as skin tones became lighter (*B* = −2.24, *SE* = 0.41), *t*(24) = 5.42, *p* < 0.001 (See Figure 10). There were no other significant interactions among the variables tested (*p* > 0.55).

#### Chroma

**Figure 11.**
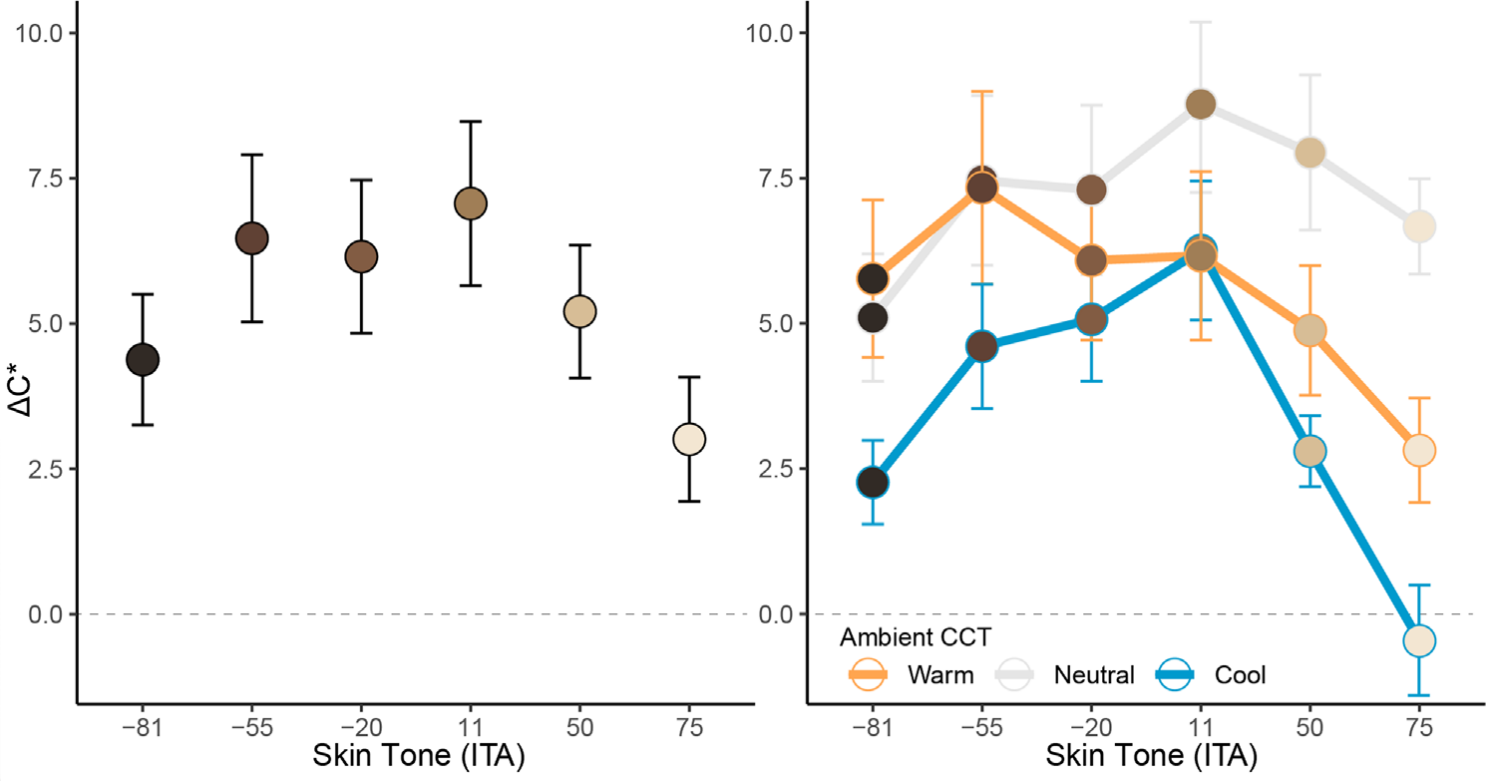
Summary (mean and SE) of the influence of skin tone (ITA) on chroma adjustments made to AR faces to match the appearance of emissive faces (left) and its interaction with ambient CCT (right) in the color-matching task. Participants increased chroma more for mid-skin tones than both very dark and very light skin tones. This pattern was more pronounced under cool ambient CCT conditions, relative to neutral and warm CCTs.

There was a significant effect of skin tone on ΔC*, *F*(5, 120) = 14.25, *p* < 0.001, with a significant quadratic trend, *F*(1, 120) = 57.37, *p* < 0.001, indicating that both very dark and very light skin tones needed less chroma adjustments than mid-skin tones (see Figure 11). There was a significant effect of ambient luminance on ΔC*, *F*(1, 24) = 10.89, *p* = 0.003, indicating that participants increased chroma more under high luminance (*M* = 5.99, *SE* = 0.79) than low luminance (*M* = 4.78, *SE* = 0.61) ambient conditions. There was a significant effect of ambient CCT on ΔC*, *F*(2, 48) = 19.31, *p* < 0.001. Participants increased chroma most for neutral CCT (*M* = 7.21, *SE* = 0.84), followed by warm CCT (*M* = 5.51, *SE* = 0.89), *t*(24) = 3.04, *p* = 0.006, which was higher than cool CCT (*M* = 3.43, *SE* = 0.50), *t*(24) = 3.22, *p* = 0.004 (see Figure 11). There was also a statistically significant interaction between skin tone and ambient CCT on ΔC*, *F*(10, 240) = 5.47, *p* < 0.001, indicating that the influence of skin tone on ΔC* varied as a function of ambient CCT. The quadratic trend of skin tone on chroma adjustments was more pronounced under cool CCT, *F*(1, 120) = 74.31, *p* < 0.001, than neutral CCT, *F*(1, 120) = 16.49, *p* < 0.001, and warm CCT, *F*(1, 120) = 10.13, *p* = 0.002 (see Figure 11 right).

#### Hue

Finally, we evaluated the extent to which participants adjusted AR stimulus hue to match emissive stimuli as close as possible. While participants adjusted hue angle (Δh°) in the experiment, we used ΔH* as the dependent variable for the subsequent analyses. ΔH* is calculated as the color difference attributable to changes in hue after removing lightness and chroma adjustments from the total color difference (Berns, 2019). As such, ΔH* is a Euclidean distance metric (as opposed to the Δh° polar metric), and is therefore more directly comparable to the other distance metrics (ΔL*, ΔC*) reported in the current work.

**Figure 12.**
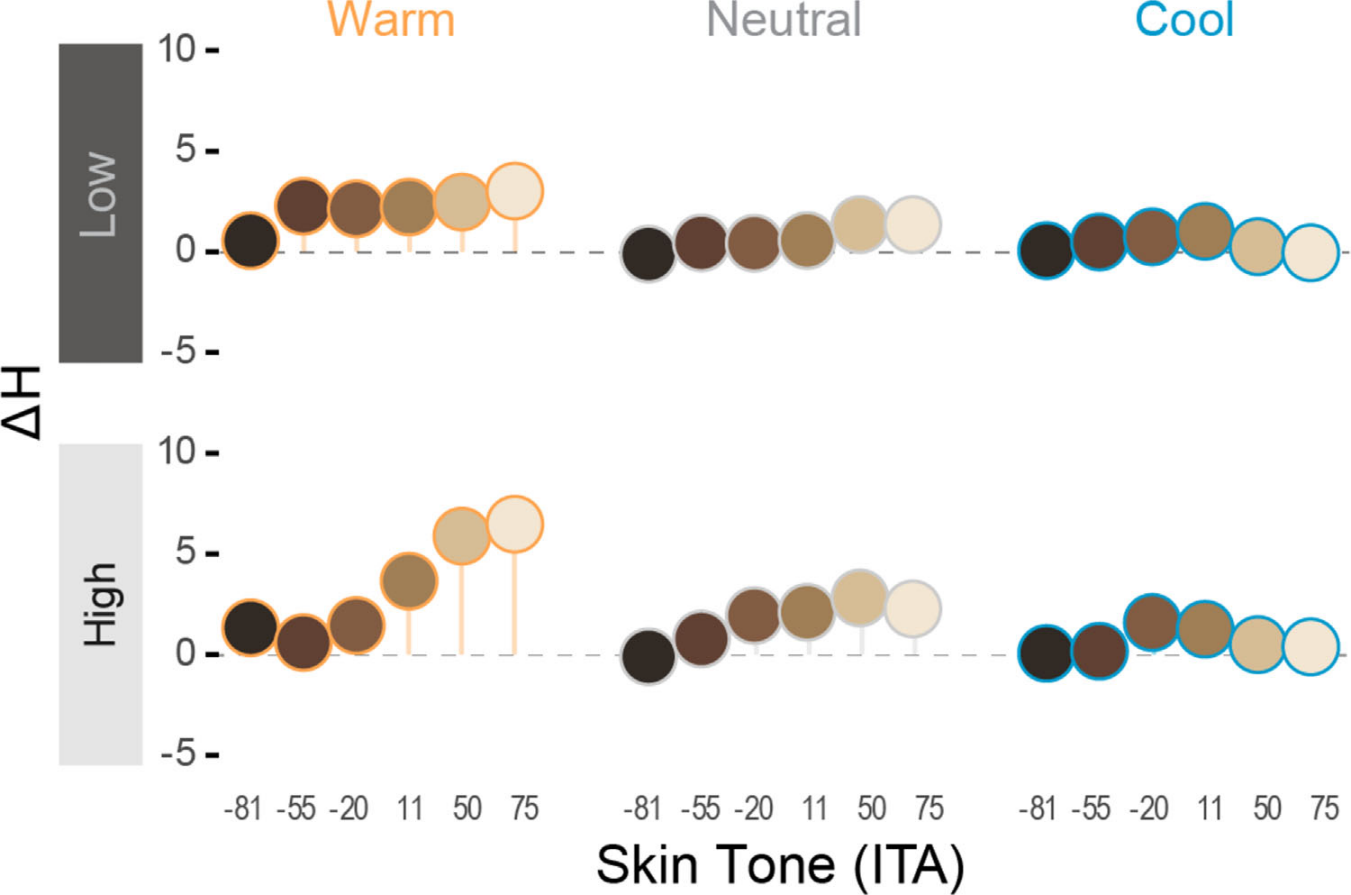
The ΔH* adjustments made for AR stimuli to match the appearance of emissive targets in the color-matching task. Participants tended to adjust hue toward the yellow direction as CCT moved from cool-to warm, although this effect was most evident for faces with lighter skin tones in high ambient luminance conditions.

**Figure 13.**
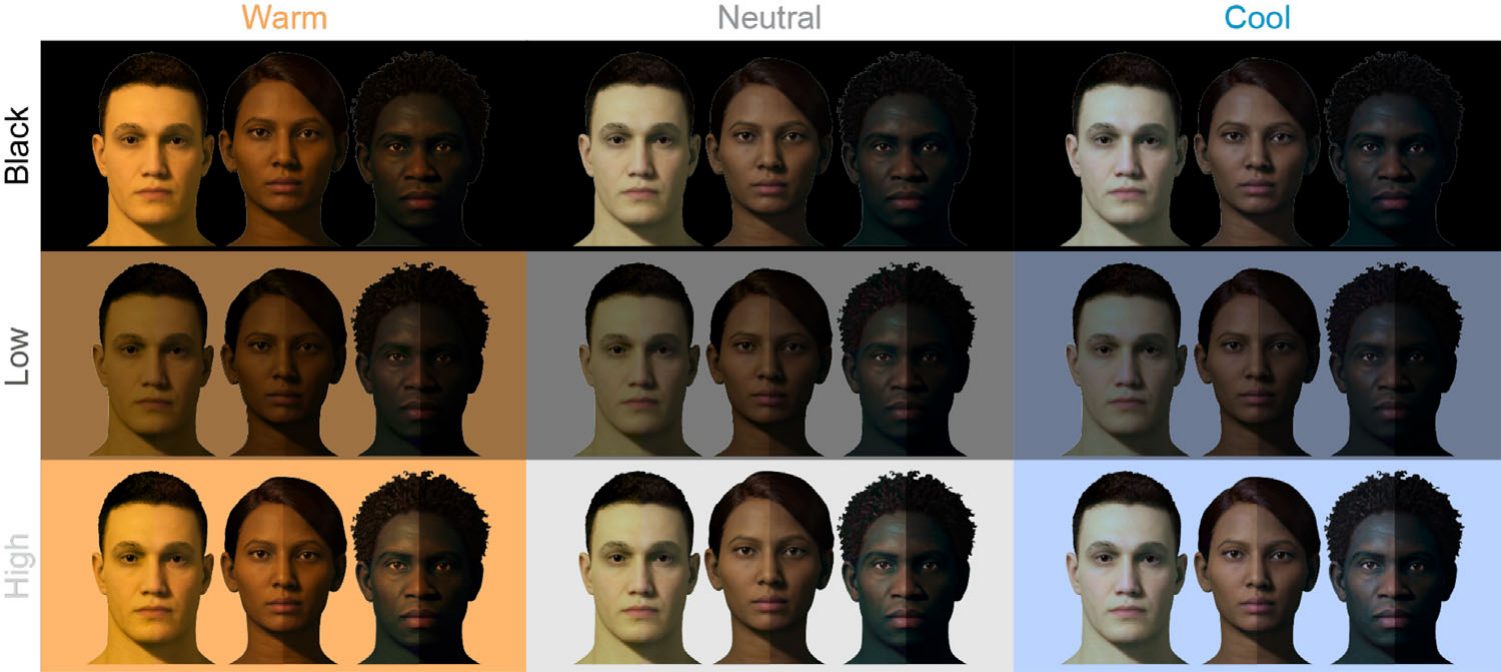
Visual approximation of mean color adjustments made to AR faces (left-bipartite) to match the appearance of emissive faces (right-bipartite), for a subset of stimuli under each ambient condition. Visual differences between bipartites are unnoticeable in the black-background (control) conditions. Lightness differences are clearly noticeable as ambient luminance increases (“Low,” “High”), particularly for darker skin tones. In those conditions, some chroma differences are noticeable for mid skin tones, and small hue differences are barely noticeable for light skin tones.

There was a significant three-way interaction between skin tone, ambient luminance, and ambient CCT on ΔH* for AR stimuli, *F*(10, 240) = 6.53, *p* < 0.001. Participants increased hue (i.e., adjusted it toward the yellow polar coordinate) as CCT moved from cool-to-warm (*B* = 1.69, *SE* = 0.33, *p* < 0.001). This increase became more pronounced as skin tones became lighter (*B* = 0.67, *SE* = 0.23, *p* = 0.005), a pattern that was more evident in the high ambient luminance condition (*B* = 1.38, *SE* = 0.39, *p* = 0.001; See Figure 12). It may be worth noting that while we found these hue adjustments to be *statistically* significant, the magnitude of some adjustments might be considered *perceptually* minor. For instance, the largest ΔH*_mean_ in the warm, highest ambient luminance condition was 6.47, so the apparent color difference between those stimuli would likely be subtle, but noticeable. However, the largest values in all other conditions were between 1.02 and 3.02, which could be considered negligible in the sense that such color differences would likely be difficult to notice based on changes to hue alone. See Figure 13 for a visual example approximating color changes made to AR faces to match the appearance of emissive faces, and Table 3 for the corresponding mean color differences (ΔE_00_).

### Match-rating task

Repeated-measures ANOVA was conducted to evaluate the extent to which participants’ adjusted AR faces matched the appearance of the emissive faces. The independent variables included skin tone (6), ambient luminance condition (3; black, low, high), and ambient CCT (3; warm, neutral, cool). The dependent variable was match-rating, standardized (*z*-scores) within participants. For each participant, raw ratings were standardized by subtracting the participant's mean rating and dividing by their standard deviation. This within-subject transformation allowed us to place all responses on a common scale while preserving individual response patterns.

There was a significant effect of skin tone on match-ratings, *F*(5, 120) = 3.58, *p* = 0.005, with a quadratic trend, *F*(1, 120) = 15.48, *p* < 0.001, indicating that participants generally perceived AR faces having both very dark and very light skin tones as poor appearance matches, relative to mid-skin tones. There was a significant effect of ambient luminance condition on match-ratings, *F*(2, 48) = 57.05, *p* < 0.001, indicating that participants generally perceived AR faces as having better appearance matches under the black background condition (*M* = 0.91, *SE* = 0.09) than under the low luminance condition (*M* = −0.28, *SE* = 0.09), *t*(24) = 7.45, *p* < 0.001, and the high luminance condition (*M* = −0.37, *SE* = 0.10), *t*(24) = 8.02, *p* < 0.001. The difference between the low and high luminance conditions was not significant, *t*(24) = 1.77, *p* = 0.090.

**Figure 14.**
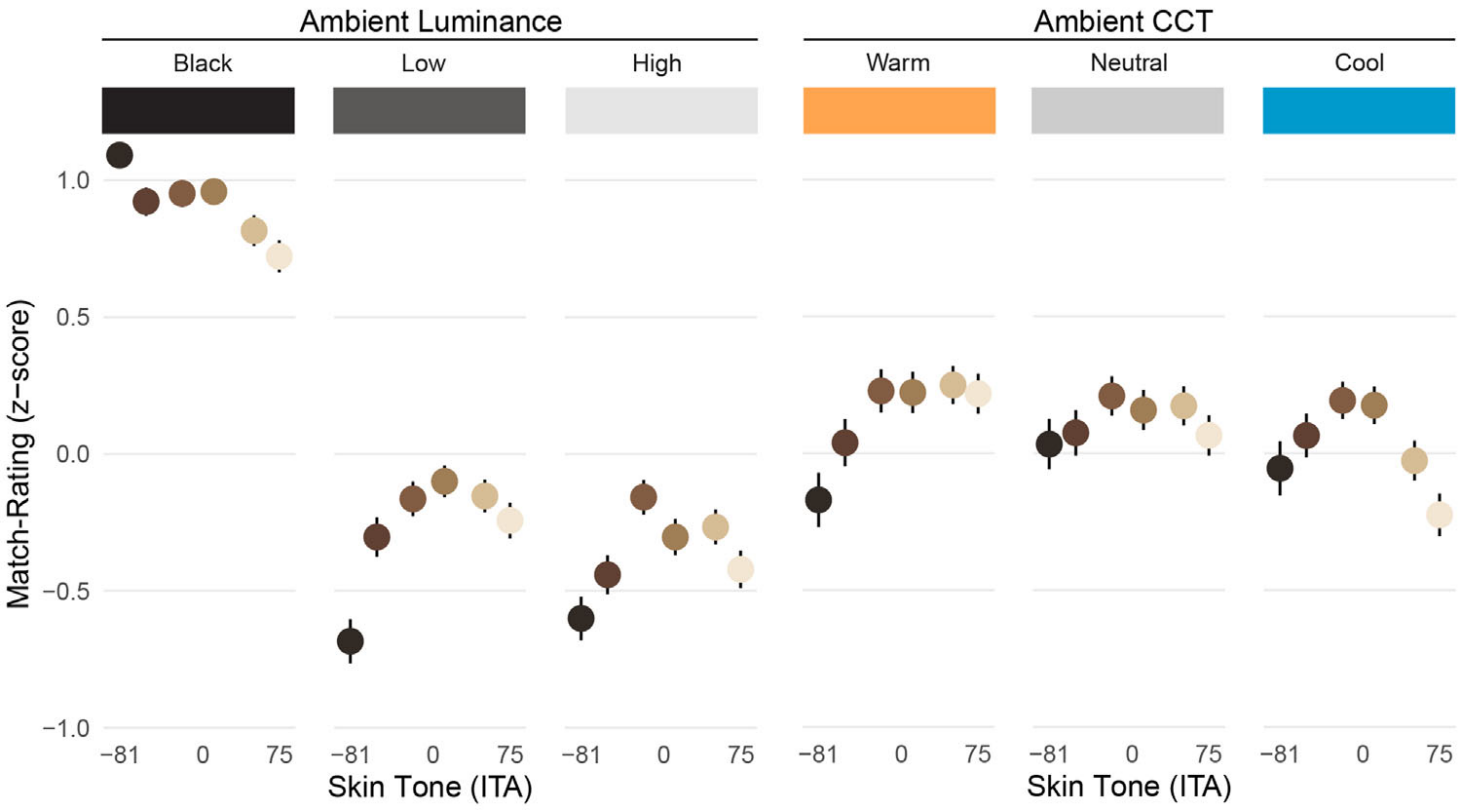
Summary (mean and SE) of the extent to which adjusted AR faces were perceived to match the appearance of emissive faces, as a function of skin tone, ambient luminance, and CCT. AR faces appeared to match very well with a black background, but then declined with additional ambient complexity. In those conditions, AR faces with the darkest skin tones tended to appear as the poorest matches, with the lightest skin tone declining as well. This pattern was consistent between low versus high ambient luminance conditions, but varied across ambient CCT conditions.

There was a significant interaction between skin tone and ambient luminance condition, *F*(10, 240) = 8.72, *p* < 0.001, showing that the quadratic pattern of skin tone on appearance matches was evident under low ambient conditions, *F*(1, 120) = 17.17, *p* < 0.001, and high ambient conditions, *F*(1, 120) = 11.87, *p* < 0.001, but not for the black background condition, *F*(1, 120) = 0.60, *p* = 0.440 (see Figure 14 left).

There was also a significant interaction between skin tone and ambient CCT condition, *F*(10, 240) = 6.07, *p* < 0.001. Subsequent analyses indicated a linear effect of skin tone on appearance matches under warm ambient conditions, *F*(1, 120) = 24.43, *p* < 0.001, a quadratic trend under cool ambient conditions, *F*(1, 120) = 19.36, *p* < 0.001, and a smaller quadratic effect under neutral ambient conditions, *F*(1, 120) = 4.28, *p* = 0.041 (See Figure 14 right).

### Preference-rating task

Repeated-measures ANOVA was conducted to evaluate how much participants liked the general appearance of their adjusted AR faces, which we refer to here as their preference for the stimuli appearance. The independent variables included skin tone (6), ambient luminance condition (3; black, low, high), and ambient CCT (3; warm, neutral, cool). The dependent variable was preference-rating, standardized (*z*-scores) within participants—using the same approach described in the Match-Rating Task section to account for individual differences in scale use.

There was a significant effect of skin tone on preference, *F*(5, 120) = 5.87, *p* < 0.001, with a significant quadratic trend, *F*(1, 120) = 26.47, *p* < 0.001. As in the previous task, participants generally least preferred AR faces having darker skin tones, with the lightest skin tone also declining in preference. There was a significant effect of ambient luminance on preference, *F*(2, 48) = 17.15, *p* < 0.001, indicating that participants preferred AR faces under the black background condition (*M* = 0.40, *SE* = 0.13) more than the low luminance (*M* = −0.29, *SE* = 0.08), *t*(24) = 3.83, *p* < 0.001, and the high luminance condition (*M* = −0.38, *SE* = 0.07), *t*(24) = 4.74, *p* < 0.001. The difference between the low and high luminance conditions was not significant (*p* = 0.192).

**Figure 15.**
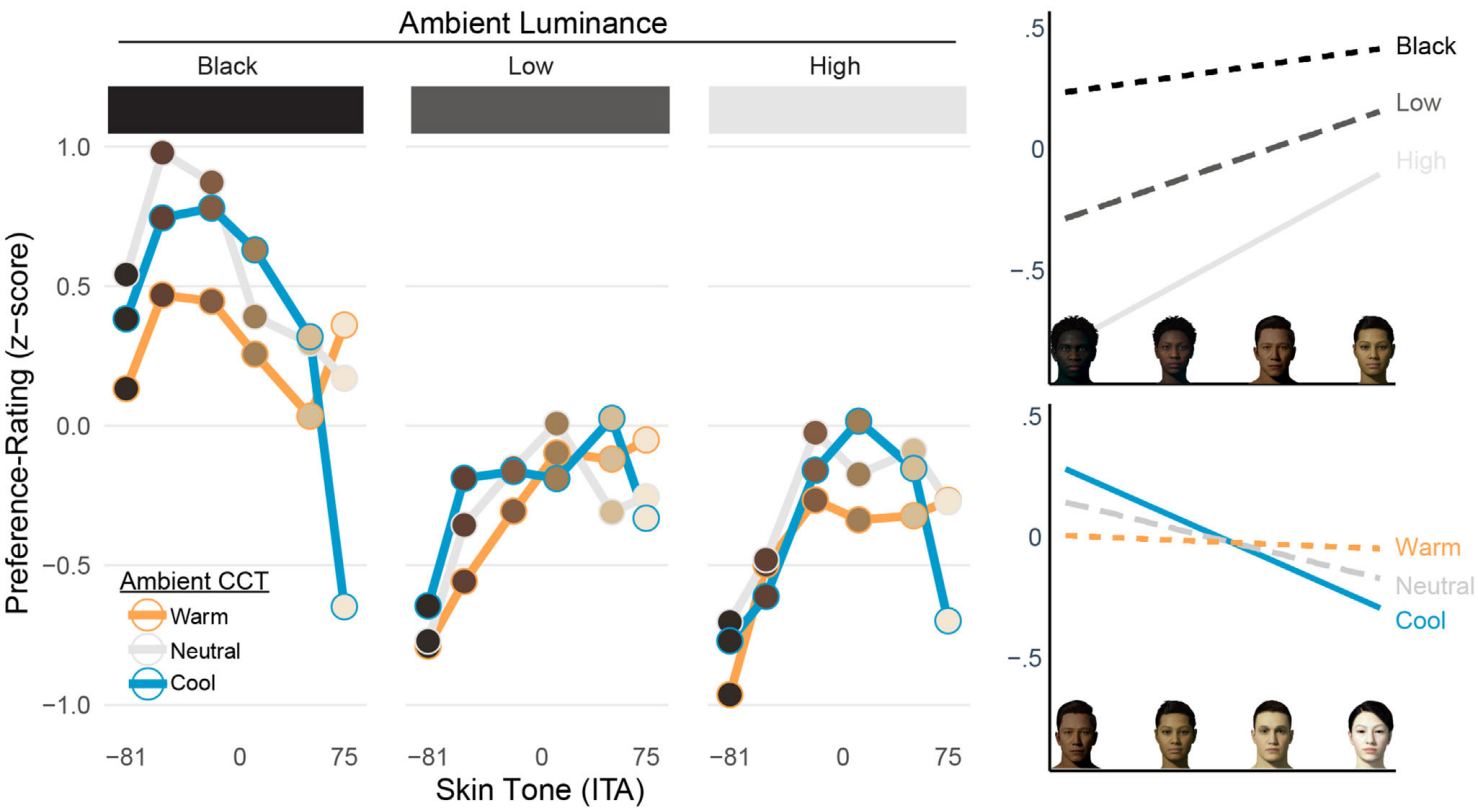
Summary of preference ratings for adjusted AR faces, as a function of skin tone, ambient luminance, and CCT (left). AR faces were generally preferred more on the black background, and preferred less with additional ambient complexity. Preferences for AR faces having darker skin tones tended to become worse as ambient luminance increased (top-right), whereas preferences for AR faces having the lightest skin tones declined more from warm-to-cool ambient conditions (bottom-right).

Additionally, there was a significant three-way interaction between the independent variables on preference ratings, *F*(20, 480) = 2.30, *p* = 0.001, suggesting that the effect of skin tone on preference varied as a function of both ambient luminance and CCT. To summarize this interaction, it appears that preferences for AR faces having darker skin tones become worse as ambient luminance increases, whereas preferences for AR faces having the lightest skin tones become worse as ambient CCT moves from warm to cool (see Figure 15).

## Discussion

Lightness was shown to be the most impactful factor in the adjustments needed for facial color appearance matching in OST-AR, relative to the other color dimensions. As expected, lightness adjustments varied largely as a function of skin tone and ambient luminance. Generally, participants needed to increase lightness more as skin tones became darker, a pattern that was exacerbated as ambient luminance increased. These observations can be explained by the additive light model of OST-AR, where darker virtual content adds much less light to the system, and is impacted more by the surrounding environment than lighter virtual content. This results in darker virtual content appearing appreciably more transparent and needing substantially greater boosts to lightness to become commensurately visible (Li & Murdoch, 2022; Zhang & Murdoch, 2021). These findings also align well with previous research showing that increases in lightness are needed more for darker facial stimuli to reduce transparency and enhance visibility, particularly under brighter ambient conditions (Doroodchi et al., 2022; Herbeck et al., 2024). It is worth noting that the applied lightness adjustments—via gamma adjustments—were intended to enhance perceptual contrast and visibility rather than to preserve strict colorimetric accuracy. Although this may lead to deviations from precise physical matches, it reflects a practical emphasis on perceived image quality in the context of OST-AR display limitations.

Chroma had the next largest influence on adjustments, after lightness. As expected, chroma adjustments were affected by changes in ambient chromaticity. Changes to initial skin chroma introduced by CATs may have necessitated the different patterns of chroma adjustment across the different ambient chromaticities. Specifically, the range of initial facial chroma across skin tones was expanded under warm CCT (and condensed under cool CCT), relative to neutral CCT. The lower initial facial chroma under cool CCT likely explains the smaller chroma adjustments observed in this condition. However, we found that chroma adjustments generally tended to be greatest under the neutral CCT condition. Although this was unexpected, it might be explained by chroma compensation from the background bleeding through the semi-transparent stimuli. The background scenes under warm and cool CCT were already more chromatic relative to the neutral CCT, which may have helped compensate for the desaturation of AR face stimuli. Additional investigations into this possibility would be worthwhile. We also found that participants increased chroma more under high ambient luminance compared to low ambient luminance conditions. In OST-AR, the added light from the background causes the virtual content in the foreground to appear more desaturated (Gabbard, Swan, Zedlitz, & Winchester, 2010; Hassani & Murdoch, 2019). To compensate, participants needed to increase chroma to a greater extent in brighter ambient conditions. Finally, we found that skin tone influenced chroma adjustments, such that mid-ITA skin tones needed more chroma increases than very light or very dark skin tones. Although we did not explicitly hypothesize this pattern, it might likely be explained by the original chroma of the faces, such that those faces also had higher chroma initially. Therefore we find it likely that additional chroma compensation was needed for those faces because they were more detrimentally impacted by desaturation in OST-AR. Gabbard et al. (2010) reported a similar phenomenon, where virtual stimuli of varying saturation became desaturated and tended to cluster around the white background color.

Hue tended to have the least impact on color adjustments to AR faces, relative to lightness and chroma. We did find that participants tended to make faces slightly yellower, particularly as ambient CCT moved from cool-to-warm, and moreso for lighter skin tones. We expected that faces would be adjusted to be yellower under warm CCT to closer fit the chromaticity of the ambient lighting. This may have been more necessary for lighter skin tones, since they were generally more opaque in OST-AR, and thus exhibited less “bleed through” of the scene chromaticity, which would have facilitated the environmental fit between scene and faces (Herbeck et al., 2024). We likewise expected to see hue adjustments in the opposite direction (toward blue) under cool CCT; however, we did not find compelling evidence to support that hypothesis in the current work. One possibility for this is that the initial chroma of faces was compressed under cool CCT, such that any subsequent changes in hue angle might be visually minor relative to the same changes made under neutral and warm CCT. Finally, while the patterns of hue adjustments described here were found to be statistically significant, we note that the numerical differences (reported as CIELAB ΔH* values) and corresponding visual differences (approximated in Figure 13) seem to indicate that such hue adjustments were likely visually negligible, in the sense that additional adjustments made to hue are unlikely to be impactful, relative to the other color dimensions, in the context of accurate facial color reproduction.

When re-assessing their responses (i.e., determining how well their adjustments actually produced a good appearance match), participants were generally satisfied under the black-background (control) condition, indicating that the OST-AR apparatus is capable of accurate facial color reproduction in the absence of any ambient complexity. Likewise, preference-ratings for participants’ adjusted AR faces largely mirrored their match-ratings. However, these perceptions greatly declined with additional ambient complexity. In those conditions, AR faces with both very dark and very light skin tones were perceived as having particularly poor matches and preferences. This aligns with our prediction that faces having darker skin tones would exhibit decreased accuracy and preferences, given the substantial adjustments needed to compensate for their diminished appearance in OST-AR. However, we also found relatively poor ratings for the lightest skin tones, which were observed even under the black background condition. One possible explanation might be that those faces may have looked less natural than the others; the lightest stimuli needed the largest amount of initial color processing when converting the MetaHuman model skin color to the target skin tones, and anecdotally some participants noted that they disliked the appearance of the lightest face in general. Thus it is possible that the perceived naturalness of those stimuli may have been reflected in their ratings, although we did not explicitly test for this possibility in the current work. Another possibility might be due the chroma compression that occurs when using the CAT to simulate cool ambient lighting, which would make the initial color of the lightest faces appear particularly unnatural. This possibility might be supported by the disparate patterns of ratings across CCT conditions, whereby the lightest faces are perceived as the poorest match under cool CCT, are perceived relatively consistently with other skin tones under neutral CCT, and perceived as among the best matches under warm CCT. Some of these findings align with previous research showing that perceptions of AR facial stimuli are detrimentally impacted when viewed against brighter backgrounds, and when evaluating darker skin tones (Herbeck et al., 2024; Peck et al., 2022). However, our findings regarding diminished perceptions of the lightest skin tones warrants further investigation. Altogether, the results from the match-rating and preference-rating tasks indicate that even after participants made colorimetric adjustments to AR faces to best match the emissive faces, the resulting appearance of the AR faces could still be improved. The severity of these ratings varied depending on combinations of skin tone, ambient luminance, and ambient CCT, pointing to the need for additional stimulus-by-environment approaches in rendering and displaying virtual faces in OST-AR.

The current work has limitations that should be considered for future work. First, the use of generated human faces as stimuli was a practical choice that allowed for more specificity in target skin tone implementation, but may have introduced potential artifacts. Specifically, the additional color processing applied in MATLAB after rendering MetaHumans to better match our target skin tones might have caused some stimuli, particularly the lightest faces, to appear unnatural, which potentially contributed to the lower preferences observed for these faces. Using real human faces as stimuli in future studies could help address this possibility. Second, the skin tones used in the experiment were based on the MST scale, which was designed to capture the natural diversity of human skin (Monk, 2023). However, human skin exhibits much greater variability than what any skin tone scale can summarize (Xiao et al., 2017). Although this approach was taken for practical experimental purposes, it does not fully reflect the richness of real-world skin tone diversity. In addition to focusing on variations in skin tone via melanin content as in the current study, future work that incorporates stimuli with greater chromaticity variations in skin tones may provide more comprehensive insights into the colorimetric adjustments needed for appearance matching in OST-AR environments. Third, although participants were instructed to focus on the skin areas of the face during the experiment, it is possible that other features, such as hair, eyes and lips, influenced their judgments to some extent. Fourth, future work may also consider testing brighter ambient conditions or outdoor settings. The specific ambient luminance conditions used in the current experiment were based partly on color gamut constraints for the AR display while trying to simulate disparate “white-point” chromaticities together with both very light and very dark facial stimuli. It would be worthwhile to explore how participants adjust colors when the ambient luminance is much higher, as it would be in other real-world, including outdoor, settings. In such scenarios, an AR display with a higher dynamic range would likely be beneficial. Fifth, to ensure precise control over ambient chromaticity, the current study employed simplified, chromatically neutral virtual environments. This design reduced interpretive ambiguity about the illumination source and avoided confounding effects from chromatic contrast. However, this came at the expense of ecological realism and may have made the environments feel less natural to participants. Future research could use more realistic scenes to simulate real-world lighting and examine how environmental complexity affects facial color perception in OST-AR. Finally, turning to participant-related factors, we did not collect quantitative measures of participants’ skin tone—only self-reported ethnicity. Because skin tone can vary widely within an ethnic category, this limits our ability to analyze potential bias based on participant skin tone. It is plausible that participants’ judgments, particularly in the preference-rating task, may have been influenced by the similarity between their own skin tone and the facial stimuli, but our current sample lacked the diversity and statistical power to rigorously test this possibility. Future studies with more balanced and diverse participant representation, along with direct measurement of their skin tone, would be helpful in exploring this possibility.

Beyond these limitations, a promising direction for future research would be to build on earlier work by assessing the influence of other image properties on color reproduction in OST-AR. Instead of globally manipulating the colorimetric dimensions of an image, as done in the current study, content-aware methods incorporating adaptive adjustments could further enhance image quality in OST-AR environments (Fukiage & Oishi, 2023; Lee & Kim, 2018; Zhang, Wang, Peng, Hua, & Bao, 2022). Another promising direction for future research on improving color reproduction in OST-AR involves hardware-based solutions, such as global ambient dimming with neutral density filters (Erickson, Bruder, & Welch, 2022; Mori, Ikeda, Plopski, & Sandor, 2018) or pixel-wise local occlusion (Cakmakci, Ha, & Rolland, 2004; Hu et al., 2024; Zhang, Hu, Kiyokawa, & Yang, 2023).

## Conclusions

Augmented reality aims to combine virtual content with one's physical surroundings, allowing them to appear together as part of an integrated environment. OST-AR approaches this integration by using transparent optics by which virtual content is added while directly viewing the physical environment. However, this additive light blending introduces challenges in color reproduction, including issues surrounding transparency and its interaction with complex ambient lighting, which disproportionally affects the appearance of faces having darker skin. Given the integral role of virtual faces in social AR applications, it is essential to better understand facial color reproduction in OST-AR to enhance realism, engagement, and accessibility with these emerging technologies. The current work assessed colorimetric adjustments needed to best reproduce OST-AR faces as a function of facial skin tone, ambient luminance, and ambient chromaticity. We found that substantial adjustments to lightness, moderate adjustments to chroma, and minor adjustments to hue were needed, whereas the relative influence of each dimension varied across skin tones and ambient lighting conditions. We also identified conditions where the resulting appearance of adjusted AR faces remained sub-optimal with respect to their perceived appearance match and preference. The current findings extend past work on OST-AR facial color reproduction by using an experimentally controlled set of skin tones that vary systematically as a function of estimated skin melanin, by using simulated ambient conditions varying in both luminance and chromaticity, and by describing the magnitude of adjustments needed with respect to perceptually orthogonal color dimensions. This work builds upon the growing body of literature concerning color appearance in extended reality environments, and highlights the importance of understanding visual perception of human faces specifically. It is expected that the current work can contribute toward improving the display of human faces in AR, and support the development of more enjoyable, engaging, and equitable social experiences in extended reality more broadly.
